# Epidemiology, antifungal susceptibility, risk factors, and mortality of persistent *candidemia* in adult patients in China: a 6-year multicenter retrospective study

**DOI:** 10.1186/s12879-023-08241-9

**Published:** 2023-06-01

**Authors:** Yanping Li, Chenghong Gu, Yuling Yang, Yinhuan Ding, Caihong Ye, Min Tang, Jinbo Liu, Zhangrui Zeng

**Affiliations:** 1 Department of Laboratory Medicine, the Affiliated Hospital of Southwest Medical University, 25 Taiping street, Luzhou, 646000 P.R. China; 2 Department of Laboratory Medicine, Zigong Fourth People’s Hospital, Zigong, 643000 P.R. China; 3 Department of Laboratory Medicine, The Second People’s Hospital of Neijiang, Neijiang, 641000 P.R. China; 4 Department of Laboratory Medicine, Luxian People’s Hospital, Luxian, 646100 Sichuan Province P.R. China; 5 Sichuan Province Engineering Technology Research Center of Clinical Diseases Molecular Diagnosis, Luzhou, 646000 P.R. China; 6 Clinical Diseases Molecular Diagnosis Key Laboratory of LuZhou, Luzhou, 646000 P.R. China

**Keywords:** Adult patients, Persistent candidemia, Epidemiology, Risk factors, Antifungal susceptibility, Mortality

## Abstract

**Background:**

Data on persistent candidemia (PC), a recognized complication of candidemia, are lacking in China. This study aimed to investigate the clinical characteristics and risk factors for the mortality of PC among adults in China.

**Methods:**

This 6-year retrospective study analyzed the prevalence, species distribution, antifungal susceptibility, risk factors, and patient mortality of PC among adults in three regional tertiary teaching hospitals in China from 2016 to 2021. We collected electronic laboratory records data of PC and non-PC patients and used the Student test or Mann–Whitney U test for a retrospective study. Logistic regression was used to identify risk factors associated with persistent candidemia.

**Results:**

The definition of PC was fulfilled by 36 patients (13.7%, 36/263). The mean age of the patients was 59.9 years (60 years for patients with PC; 59.8 years for those with non-PC; *P* > 0.05) and 131 (60.1%) were men [16 with PC (44.4%), 115 with non-PC (63.2%), *P* < 0.05]. The mean annual incidence was 0.15/1000 admissions (including PC 0.03/1000 admissions vs. non-PC 0.12/1000 admissions, *P* < 0.05). *Candida parapsilosis* (14/36, 38.9%) and *Candida albicans* (81/182, 44.5%) were the predominant pathogens in patients with PC and non-PC, respectively. Most isolates were susceptible to flucytosine (99.0%) and amphotericin B (99.5%), and the activity of antifungal agents against *Candida* species was not statistically significantly different between patients with PC and non-PC (*P* > 0.05). The 30-day mortality rate was 20.2% (16.7% with PC vs. 20.9% with non-PC, *P* > 0.05). Multivariable regression analysis showed that use of broad-spectrum antibiotics (odds ratio (OR), 5.925; 95% confidence interval (CI), 1.886–18.616, *P* = 0.002), fluconazole (OR, 3.389; 95% CI, 1.302–8.820, *P* = 0.012) and *C. parapsilosis* infection (OR, 6.143; 95% CI, 2.093–18.031, P = 0.001) were independent predictors of PC, sex (male) (OR, 0.199; 95% CI, 0.077–0.518, P = 0.001) was the protective factor for PC. Respiratory dysfunction (OR, 5.763; 95% CI, 1.592–20.864, *P* = 0.008) and length of hospital stay(OR, 0.925; 95% CI, 0.880–0.973, P = 0.002) were independent predictors of 30-day mortality in patients with non-PC. *C. tropicalis* bloodstream infection (OR, 12.642; 95% CI, 1.059–150.951; *P* = 0.045) was an independent predictor of 30-day mortality in patients with PC.

**Conclusions:**

The epidemiological data of patients with PC and non-PC were different in the distribution of *Candida* species, the mean annual incidence and independent predictors of 30-day mortality. Flucytosine and amphotericin B could be used as first-choice drugs in the presence of PC infections.

**Supplementary Information:**

The online version contains supplementary material available at 10.1186/s12879-023-08241-9.

## Background

Candidemia is the most common fungal disease among hospitalized patients worldwide and is defined as a condition in which at least one blood culture appears positive for the *Candida* species [1]. The morbidity and mortality of Candidemia in adults were higher than those in children in the last decades, however, the epidemiology of candidemia has now changed, and the incidence of candidemia in infants and children was gradually increased. It has been reported that the incidence of candidemia was more than 90% in the neonatal intensive care unit [2]. Meanwhile, the outbreak of *Candida auris* infections [3] and persistent candidemia (PC) have also brought serious challenges to the treatment of candidemia. PC is defined as the isolation of the same *Candida* species from positive blood culture for ≥ 5 days after the initiation of antifungal therapy [4]. It is an increasingly recognized complication of candidemia [5]. Previous studies reported that 8–15% of patients with candidemia developed PC [6]; meanwhile, PC was associated with significant mortality, which was as high as 20–50% [4, 7]. Some studies reported that PC was associated with the biofilm production of *Candida* species [8, 9] and antifungal resistance [10]. The other main risk factors for PC included underlying disease status (e.g., hematological malignancies), low serum levels of drugs, endovascular infection, deep-tissue abscesses, metastatic infection foci, ineffective empirical treatment, infections associated with prosthetic materials, central venous catheterization (CVC)-related infection, total parenteral nutrition, hemodialysis and abdominal surgery [11, 12].

PC often leads to poor clinical outcomes. However, only a few studies have reported on PC in adults and neonates worldwide. National or multicenter studies in patients with PC infection are almost absent in most countries and regions in the world, bringing in difficulties for preventing and treating PC. Only a few studies have reported on PC infection in infants in China. The multicenter study data of PC in adult patients are lacking in China. Therefore, we performed a 6-year retrospective study to evaluate the epidemiology, antifungal susceptibility, risk factors and mortality of PC among all adult patients in three tertiary teaching hospitals in three different cities of China.

## Methods

### Study design

We conducted a retrospective observational study of electronic laboratory records of persistent candidemia patients from the Affiliated Hospital of Southwest Medical University (AHSWMU; Luzhou, China), Zigong Fourth People’s Hospital (ZGFPH; Zigong, China) and the Second People’s Hospital of Neijiang (SPHNJ; Neijiang, China) from January 2016 to December 2021. The AHSWMU is a 3200-bed tertiary care teaching hospital with 43 wards and approximately 130,000 annual admissions, the ZGFPH is a 1600-bed tertiary care teaching hospital with 32 wards and approximately 70,000 annual admissions, the SPHNJ is a 1500-bed tertiary care teaching hospital with 38 wards and approximately 45,000 annual admissions.

### Data collection

The fungal specimen data were collected from patients with candidemia admitted to the AHSWMU, ZGFPH and SPHNJ from January 2016 to December 2021. All data were collected from electronic medical records. The following data were retrospectively collected from all adult patients: demographic characteristics, underlying comorbidities, Candida species, susceptibility to antifungal agents, use of broad-spectrum antibiotics, antifungal agents and mortality. Data on the following risk factors associated with candidemia were also collected: indwelling central vascular catheter, mechanical ventilation, systemic corticosteroid treatment (a dose equivalent to prednisone 10 mg/day for at least 14 days), total parenteral nutrition, malnutrition, chemotherapy, hemodialysis, abdominal surgery, intensive care unit (ICU) admission, neutropenia (absolute neutrophil count < 500 cells/µL), concomitant bacterial infections, septic shock, hemorrhagic shock, broad-spectrum antibiotic use, prophylaxis antifungal therapy and treatment with antifungal agents. The study protocol was approved by the Clinical Research Ethics Committee of the Affiliated Hospital of Southwest Medical University (Project No. KY2020043). The need for informed consent was waived by the Clinical Research Ethics Committee of the Affiliated Hospital of Southwest Medical University. All experiments were performed in accordance with the study protocol in three hospitals.

### Inclusion/exclusion criteria

The diagnostic criteria of candidemia were based on the guidelines for diagnosing and treating candidiasis: the expert consensus issued by the Chinese Adult Candidiasis Diagnosis and Management Expert Consensus Group [13]. These criteria were also in accordance with the European Society of Clinical Microbiology and Infectious Diseases ESCMID* guidelines for the diagnosis and management of *Candida* diseases 2012 [14, 15] and the Infectious Diseases Society of America Clinical Practice Guidelines for the Management of Candidiasis: 2016 Update [16]. All patients aged ≥ 18 years who presented to the three tertiary hospitals with candidemia from 2016 to 2021 were investigated; only the first episode was included in our analysis. Patient cultures with two or more *Candida* species were excluded from the analysis. PC was defined as the isolation of the same *Candida* species from positive blood culture for ≥ 5 days after the initiation of an antifungal therapy, and non-PC was defined as all other candidemia cases other than the persistent ones, with at least one negative blood culture between two positive blood culture results.

**Table 1:** Patient characteristics and incidence (episode/1000 admission)

	Total patients^&^	Persistent	Non-persistent	P*
	(*n* = 218)	(*n* = 36)	(*n* = 182)	
Age, years, mean (SD)	59.9(16.7)	60.0(15.0)	59.8(17.1)	0.950
**Gender (male:female)**	**131: 87**	**16:20**	**115:67**	**0.036**
**Length of hospital stay(days)(SD)**	**38.4(54.6)**	**58.3(74.7)**	**34.5(49.0)**	**0.016**
**Underlying comorbidities (n, %)**				
Gastrointestinal perforation	54 (24.8)	10(27.8)	44(24.2)	0.647
Respiratory dysfunction^a^	100 (45.9)	14 (38.9)	86(47.3)	0.375
Pulmonary infection	138(63.3)	25 (69.4)	113(62.1)	0.403
Cardiovascular disease	118 (54.1)	21(58.3)	97 (53.3)	0.579
Neurological diseases	74 (33.9)	17(47.2)	57(31.3)	0.066
Gastrointestinal diseases^b^	107(49.1)	17(47.2)	90(49.5)	0.761
Chronic/acute liver disease	85(39.0)	12(33.3)	73(40.1)	0.446
Chronic/acute renal failure^c^	**118(54.1)**	**13(36.1)**	**105 (57.7)**	**0.018**
Solid tumour	33(15.1)	5(13.9)	28(15.4)	0.819
Haematological malignancy	19 (8.7)	2 (5.6)	17(9.3)	0.486
hypertension	33(15.1)	8 (22.2)	25 (13.7)	0.194
Diabetes mellitus	66(30.3)	7(19.4)	59 (32.4)	0.122
Hematologic (nonmalignant)	66(30.3)	14 (38.9)	52 (28.6)	0.218
HIV/AIDS	6 (2.8)	0(0.0)	6 (3.3)	0.269
Severe trauma	27(12.4)	6 (16.7)	21 (11.5)	0.393
**Risk factors (n, %)**				
**Presence of CVC**^**d**^	**105(48.2)**	**23(63.9)**	**82(45.1)**	**0.039**
Other invasive catheters	68(31.2)	14(38.9)	54(29.7)	0.275
Mechanical ventilation	100(45.9)	19 (52.8)	81(44.5)	0.363
Receipt of corticosteroids^e^	13 (6.0)	2(5.6)	11(6.0)	0.910
Total parenteral nutrition	63(28.9)	13(36.1)	50(27.5)	0.296
Malnutrition	108(49.5)	21(58.3)	87(47.8)	0.248
Chemotherapy	33(15.1)	5(13.9)	28(15.4)	0.819
Hemodialysis	45(20.6)	9(25.0)	36(19.8)	0.480
Abdominal surgery^f^	52 (23.9)	10 (27.8)	42 (23.1)	0.545
ICU	83(38.1)	16 (44.4)	67 (36.8)	0.389
Neutropenia^g^	34(15.6)	5 (13.9)	29(15.9)	0.757
Electrolyte abnormalities	65(29.8)	9(25.0)	56(30.8)	0.489
Concomitant bacterial infections	78(35.8)	17 (47.2)	61 (33.5)	0.117
Septic shock	68 (31.2)	12 (33.3)	56(30.8)	0.762
Hemorrhagic shock	7(3.2)	0(0.0)	7(3.8)	0.232
**Therapy**				
Broad-spectrum antibiotics	**139(63.8)**	**29 (80.6)**	**110 (60.4)**	**0.022**
Prophylaxis antifungal therapy(FCA)	61 (28.0)	13(36.1)	48(26.4)	0.234
**After positive blood culture**				
Amphotericin B	17(7.8)	1(5.6)	16(8.8)	0.219
Capofungin	27(12.4)	3(8.3)	24(13.2)	0.419
**Fluconazole**	**61(28.0)**	**15(41.7)**	**46(25.3)**	**0.045**
Voriconazole	68(31.2)	12(33.3)	56(30.8)	0.762
Capofungin + Fluconazole	17(7.8)	1(5.6)	16(8.8)	0.219
Capofungin + Amphotericin B	6(2.8)	0(0.0)	6(3.3)	0.269
**Capofungin + Voriconazole**^**#**^	**5(2.3)**	**3(8.3)**	**2(1.1)**	**0.008**
Fluconazole + Voriconazole	13(6.0)	1(2.8)	11(6.0)	0.432
Fluconazole + Amphotericin B	5(2.3)	0(0.0)	5(2.7)	0.314
***Albicans***vs.***non-albicans Candida spp***.				0.001
* C. albicans*	**86(39.4)**	**5(13.9)**	**81(44.5)**	**0.001**
non-*C. albicans*	132(60.6)	31(86.1)	101(55.5)	0.001
***Candida*species**				
***C. albicans***	**86(39.4)**	**5(13.9)**	**81(44.5)**	**0.001**
* C. glabrata*	44(20.2)	6(16.7)	38(20.9)	0.565
* C. Tropicalis*	42(19.3)	7(19.4)	35(19.2)	0.976
*** C. Parapsilosis***	**34(15.6)**	**14(38.9)**	**20(11.0)**	**< 0.001**
*** C. kruseii***	**5(2.3)**	**3(8.3)**	**2(1.1)**	**0.008**
other *Candida* species	7(3.2)	1(2.8)	6(3.3)	0.872
**Wards**				
Medical wards	89(40.8)	11(30.6)	78(42.9)	0.170
Surgical wards	63(28.9)	13(36.1)	50(27.5)	0.296
ICU	66(30.3)	12(33.3)	54(29.7)	0.662
Outcome				
7 days death	22(10.1)	1(2.8)	21(11.5)	0.111
30 days death	44(20.2)	6(16.7)	38(20.9)	0.565
Death	57(26.1)	11(30.6)	46(25.3)	0.510
Incidence (n,episodes/1,000 admissions)				
2016	29(0.16)	5(0.03)	24(0.13)	-
2017	29(0.13)	6(0.03)	23(0.1)	-
2018	34(0.14)	4(0.02)	30(0.12)	-
2019	46(0.17)	10(0.04)	36(0.13)	-
2020	37(0.15)	4(0.02)	33(0.13)	-
2021	43(0.15)	7(0.03)	36(0.12)	-
Mean annual incidence	218(0.15)	36(0.03)	182(0.12)	-

*Statistical results of demographic characteristics of Persistent and Non-persistent candidemia patients

^&^Because the number was very small, the result of multivariable logistic regression analysis is unreliable

^&^Includes persistent and non-persistent candidemia patients

^a^ Includes the following diseases: chronic obstructive pulmonary disease and acute respiratory distress syndrome

^b^ Includes the following diseases: cholecystitis, pancreatitis and peritonitis

^c^ Chronic/Acute renal failure is the permanent or sudden and often temporary loss of kidney function with N waste retention and hypourocrinia

^d^ CVC = central venous catheter

^e^a dose equivalent to the prednisone dosage of 0.3 mg/kg/day for at least 14 days

^f^ including gastrointestinal perforations, severe acute pancreatitis and complex ventral hernia

^g^ Neutropenia is the absolute neutrophil count, that is < 500 cells/µl

### Microorganism identification and antifungal susceptibility

According to the manufacturer’s instructions, blood(10ml) was inoculated into both aerobic and anaerobic BacT/AlerT 3D vials (bioMérieux, France). All positive cultures were manually sampled and inoculated into CHROMagar Candida medium (CHROMagar Company, France) to ensure viability and purity. The identification of all species was confirmed by a Vitek-2 system (bioMérieux, Marcy L’Etoile, France) at SPHNJ and Microflex LT (Bruker Diagnostics Inc., USA) matrix-assisted laser-desorption/ionization time-of-flight mass spectrometry system at AHSWMU and ZGFPH.

Antifungal susceptibility tests for fluconazole (FCA), itraconazole (ITR), voriconazole (VRC), flucytosine (5-FC) and amphotericin B (AMB) were performed for all *Candida* strain isolates using an ATB FUNGUS 3 kit (bioMérieux, France) in all the three hospitals. The minimal inhibitory concentrations of the antifungal agents were judged by visualization in our laboratory according to the manufacturer’s instructions. The quality control strains were *Candida parapsilosis* ATCC 22,019 and *C. krusei* ATCC 6258. The results were interpreted using the Clinical and Laboratory Standards Institute M27-A3 microbroth dilution method [17].

### Statistical analyses

The data were analyzed using Microsoft Excel (version 2019, WA, USA) and IBM SPSS software version 26 for Windows (IBM, NY, USA). The categorical data were compared using chi-square or Fisher’s exact tests. The continuous data were analyzed using the Student *t* test or Mann–Whitney *U* test. Multivariable logistic regression analysis was performed to identify independent predictors of PC and 30-day hospital mortality. Odds ratios (ORs) and 95% confidence intervals (CIs) were calculated. Biologically plausible variables with a *P* value < 0.1 according to the univariate analyses were included in the multiple logistic regression model. Statistical significance was determined using two-tailed tests, and *P* < 0.05 was considered statistically significant [4].

**Fig. 1:**
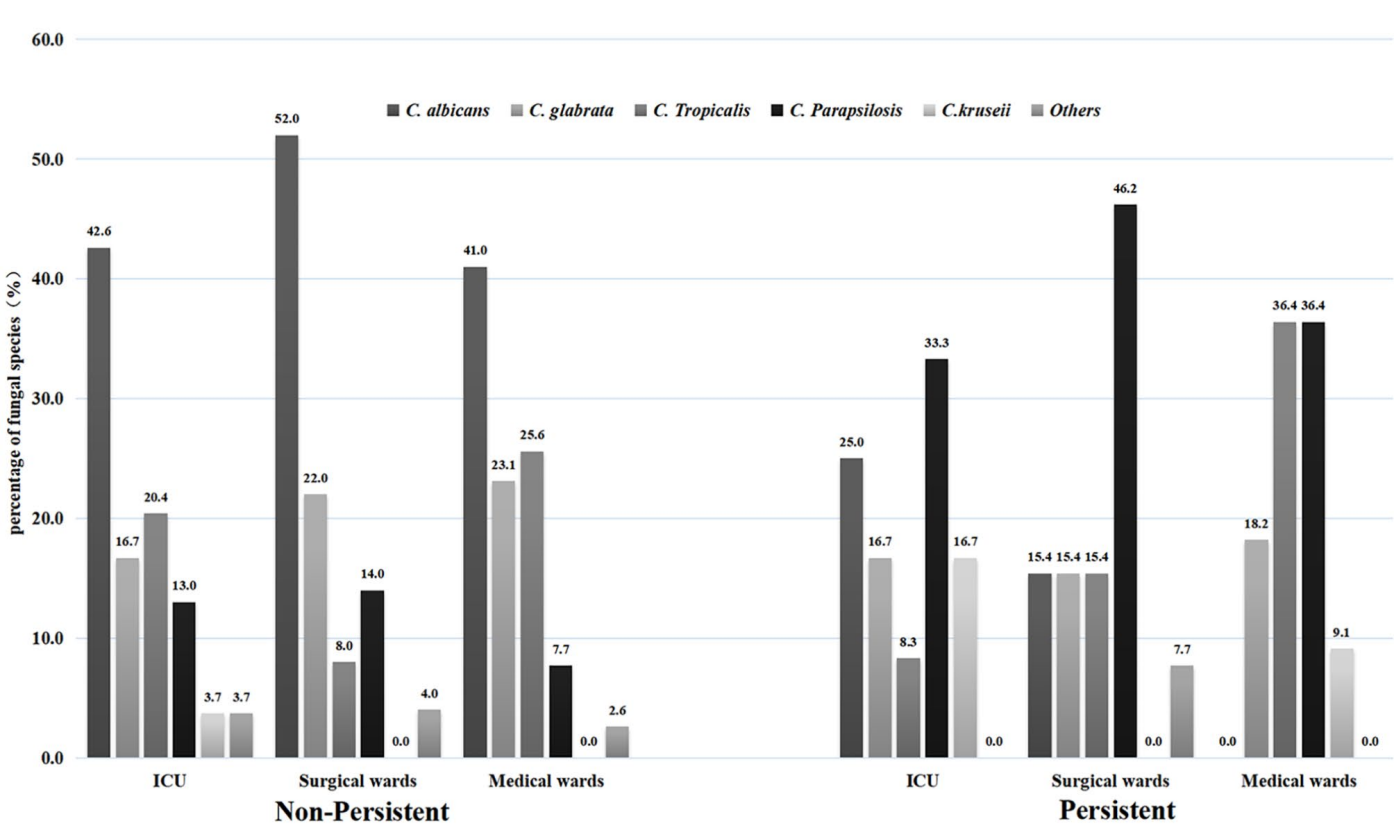
Distribution of the fungal species in adult patients with PC and Non-PC according to different wards FootNote: *Others include *C. guilliermondii* (2), *C. famata* (2), *C. dubliniensis* (1), *C. haemulonii* (1) and *C. inconspicua* (1)

**Table 2:** In vitro antifungal susceptibility testing of 203 clinical isolates into 5 antifungal agents

Species (No of isolates)	Antifungal agent	Resistant n(%)			
		Non- PC(167)	PC(36)	total	P^c^
*C. albicans* (77)	Amphotericin B	0	0	0^b^	-
	Flucytosine	1(1.4)	0	1(1.3)^b^	0.791
	Fluconazole	9(12.5)	2(40.0)	11(14.3)	0.089
	Itraconazole	15(20.8)	2(40.0)	17(22.1)^b^	0.318
	Voriconazole	13(18.1)	1(20.0)	14(18.2)^b^	0.913
* C. glabrata* (40)	Amphotericin B	0	0	0^b^	0.078
	Flucytosine	0	0	0^b^	-
	Fluconazole	2(5.9)	0	2(5.0)	0.542
	Itraconazole	4(11.8)	0	4(10.0)^b^	0.376
	Voriconazole	0	0	0^b^	-
*C.tropicalis* (41)	Amphotericin B	1(2.9)	0	1(2.4)^b^	0.646
	Flucytosine	0	0	0^b^	-
	Fluconazole	12(35.3)	3(42.9)	15(36.6)^b^	0.705
	Itraconazole	12(35.3)	3(42.9)	15(36.6)^b^	0.705
	Voriconazole	13(38.2)	3(42.9)	16(39.0)^b^	0.819
* C. parapsilosis* (33)	Amphotericin B	0	0	0	-
	Flucytosine	0	0	0	-
	Fluconazole	0	0	0	-
	Itraconazole	0	0	0	-
	Voriconazole	0	0	0	-
*C. krusei* (5)	Amphotericin B	0	0	0	-
	Flucytosine	1(50.0)	0	1(20.0)^b^	0.171
	Fluconazole^a^	-	-	-	
	Itraconazole	0	2(66.7)	2(40.0)^b^	0.136
	Voriconazole	0	1(33.3)	1(20.0)^b^	0.361
Others*(7)	Amphotericin B	0	0	0	-
	Flucytosine	0	0	0	-
	Fluconazole	0	0	0	-
	Itraconazole	0	0	0	-
	Voriconazole	0	0	0	-
All of isolates (204)	Amphotericin B	1(0.6)	0	1(0.5)	0.642
	Flucytosine	2(1.2)	0	2(1.0)	0.509
	Fluconazole^a^	23(13.9)	5(15.2)	28(14.1)	0.285
	Itraconazole	31(18.5)	7(19.4)	38(18.6)	0.902
	Voriconazole	26(15.5)	5(13.9)	31(15.2)	0.799

MIC: minimal inhibitory concentration, PC: persistent candidemia

^a^Resistance rate was based on the intrinsic resistance of *C*. *krusei* and did not follow the actual MICs.

^b^The breakpoints of *Candida* spp. according to the manufacturer’s instructions for the ATB FUNGUS 3 system

^c^The difference in resistance rate between non-PC and PC was analyzed by chi-square test

*Others include *C. guilliermondii* (2), *C. famata* (2), *C. dubliniensis* (1), *C. haemulonii* (1) and *C. inconspicua* (1)

## Results

A total of 263 distinct candidemia episodes were identified during our study period. The definition of PC was fulfilled by 36 patients (13.7%, 36/263), and 182 patients (69.2%, 182/263) had non-PC. The mean age of the patients was 59.9 years (60 years for PC, 59.8 years for non-PC, *P* > 0.05), and 131 (60.1%) were men [16 with PC (44.4%), 115 with non-PC (63.2%), *P* < 0.05]. Most PC episodes were diagnosed in surgical wards (13, 36.1%), and most non-PC episodes in medical wards (78, 42.9%). The detailed data from the three hospitals are shown in Supplementary Table S1. Most of the patients with PC and non-PC had one or more comorbidities. Pulmonary infection (69.4%), cardiovascular disease (58.3%), neurological diseases (47.2%) and gastrointestinal diseases (47.2%) were the most common underlying comorbidities in patients with PC, whereas pulmonary infection (62.1%), chronic/acute renal failure (57.7%) and cardiovascular disease (53.3%) were the most common underlying comorbidities in those with non-PC. Moreover, the most common underlying conditions documented before PC and non-PC were prior exposure to broad-spectrum antibiotics (80.6% and 60.4%, respectively), CVC (63.9% and 45.1%, respectively), malnutrition (58.3% and 47.8%, respectively), mechanical ventilation (52.8% and 44.5%, respectively), concomitant bacterial infections (47.2% and 33.5%, respectively), ICU admission (44.4% and 36.8%, respectively) and total parenteral nutrition (36.1% and 27.5%, respectively). In total, 13 (36.1%, 13/36) patients with PC had received prophylactic antifungal therapy with FCA, and patients with non-PC accounted for 26.4% (48/182) of the total. Except for chronic/acute renal failure, no statistically significant differences were found in the underlying comorbidities between patients with PC and non-PC. Moreover, no statistically significant differences in the number of underlying conditions were found between patients with persistent and non-PC (except for CVC). After the positive result of blood culture, VRC became the most important antifungal drug. The demographic and clinical characteristics of the patients are summarized in Table 1.

**Table 3:** Factors associated with 30-day mortality by univariate analysis in adult patients with candidaemia

Variable	Non- Persistent candidemia 30-days outcome		*P*-value	Persistent candidemia 30-days outcome		*P*-value	total patients* 30-days outcome		*P*-value
	Survived (*n* = 144)	Died (*n* = 38)		Survived (*n* = 30)	Died (*n* = 6)		Survived (*n* = 174)	Died (*n* = 44)	
**Demographics**									
**Age (SD) years**	**58.5(17.1 )**	**65.1(16.1)**	**0.033**	58.4 (15)	68(13.2)	0.148	**58.5 (16.8 )**	**65 0.5( 15.6)**	**0.012**
Gender (male:female)	93:51	22:16	0.447	14:16	2:4	0.549	107:67	24:20	0.400
**Length of hospital stay(days)(SD)**	**40.3(53.3)**	**12.2(11.1)**	**0.002**	64.8(80.4)	25.7(7.7)	0.247	**44.6(59.3)**	**14.1(11.6)**	**0.001**
**Underlying comorbidities (*n***, **%)**									
Gastrointestinal perforation	33(22.9)	11(28.9)	0.440	8(26.7)	2(33.3)	0.739	41(23.6)	13(29.5)	0.412
Respiratory dysfunction^a^	**55(38.2)**	**31(81.6)**	**< 0.001**	11(36.7)	3(50.0)	0.541	**66(37.9)**	**34(77.3)**	**< 0.001**
Pulmonary infection	86(59.7)	27(71.1)	0.200	21(70.0)	4(66.7)	0.871	107(61.5)	31(70.5)	0.271
Cardiovascular disease	**63(43.8)**	**34(89.5)**	**< 0.001**	19(63.3)	2(33.3)	0.174	**82(47.1)**	**36(81.8)**	**< 0.001**
Neurological diseases	42(29.2)	15(39.5)	0.223	12(40.0)	5(83.3)	0.052	54(31.0)	20(45.5)	0.071
Gastrointestinal diseases^b^	72(50.0)	18(47.4)	1.000	15(50.0)	2(33.3)	0.455	87(50.0)	20(45.5)	0.788
Chronic/acute liver disease	59(41.0)	14(36.8)	0.644	10(33.3)	2(33.3)	1.000	69(39.7)	16(36.4)	0.689
Chronic/acute renal failure^c^	**77(53.5)**	**28(73.7)**	**0.025**	12(40.0)	1(16.7)	0.277	89(51.1)	29(65.9)	0.079
Solid tumour	25(17.4)	3(7.9)	0.150	5(16.7)	0(0)	0.281	30(17.2)	3(6.8)	0.085
Haematological malignancy	13(9.0)	4(10.5)	0.791	1(3.3)	1(16.7)	0.193	14(8.0)	5(11.4)	0.509
Hypertension	22(15.3)	3(7.9)	0.240	11(36.7)	3(50.0)	0.541	27(15.5)	6(13.6)	0.410
Diabetes mellitus	44(30.6)	15(39.5)	0.296	5(16.7)	2(33.3)	0.346	49(28.2)	17(38.6)	0.177
Hematologic (nonmalignant)	39(27.1)	13(34.2)	0.387	11(36.7)	3(50.0)	0.541	50(28.7)	16(36.4)	0.325
HIV/AIDS	5(3.5)	1(2.6)	0.796	0(0)	0(0)	-	5(2.9)	1(2.3)	0.828
Severe trauma	19(13.2)	2(5.3)	0.173	4(13.3)	2(33.3)	0.230	23(13.2)	4(9.1)	0.458
**Risk factors (n, %)**									
Presence of CVC^d^	64(44.4)	18(47.4)	0.747	18(60.0)	5(83.3)	0.277	82(47.1)	23(52.3)	0.542
Other invasive catheters	**49(34.0)**	**5(13.2)**	**0.012**	12(40.0)	2(33.3)	0.760	**61(35.1)**	**7(15.9)**	**0.014**
Mechanical ventilation	**56(38.9)**	**25(65.8)**	**0.003**	14(46.7)	5(83.3)	0.101	**70(40.2)**	**30(68.2)**	**0.001**
Receipt of corticosteroids^e^	9(6.3)	2(5.3)	0.820	1(3.3)	1(16.7)	0.193	10(5.7)	3(6.8)	0.789
Total parenteral nutrition	**31(21.5)**	**19(50.0)**	**< 0.001**	11(36.7)	2(33.3)	0.877	**42(24.1)**	**21(47.7)**	**0.002**
Malnutrition	73(50.7)	14(36.8)	0.128	17(56.7)	4(66.7)	0.650	90(51.7)	18(40.9)	0.200
Chemotherapy	24(16.7)	4(10.5)	0.351	4(13.3)	1(16.7)	0.829	28(16.1)	5(11.4)	0.434
Hemodialysis	25(17.4)	11(28.9)	0.111	8(26.7)	1(16.7)	0.606	33(19.0)	12(27.3)	0.224
Abdominal surgery^f^	37(25.7)	5(13.2)	0.103	7(23.3)	3(50.0)	0.183	44(25.3)	8(18.2)	0.323
ICU^g^	49(34.0)	18(47.4)	0.129	19(63.3)	5(83.3)	0.343	68(39.1)	23(52.3)	0.113
Neutropenia^h^	25(17.4)	4(10.5)	0.306	4(13.3)	1(16.7)	0.829	29(16.7)	5(11.4)	0.386
Electrolyte abnormalities	48(33.3)	8(21.1)	0.145	8(26.7)	1(16.7)	0.606	56(32.2)	9(20.5)	0.129
Concomitant bacterial infections	**40(27.8)**	**21(55.3)**	**0.001**	15(50.0)	2(33.3)	0.455	**55(31.6)**	**23(52.3)**	**0.011**
Septic shock	**32(22.2)**	**24(63.2)**	**< 0.001**	6(20.0)	3(50.0)	0.121	**38(21.8)**	**27(61.4)**	**< 0.001**
Hemorrhagic shock	7(4.9)	0(0)	0.166	0(0)	0(0)	-	7(4.0)	0(0)	0.176
**Therapy(*n***, **%)**									
Broad-spectrum antibiotics	**80(55.6)**	**30(78.9)**	**0.009**	25(83.3)	4(66.7)	0.346	**105(60.3)**	**34(77.3)**	**0.037**
Prophylaxis antifungal therapy	37(25.7)	11(28.9)	0.686	11(36.7)	2(33.3)	0.877	48(27.6)	13(29.5)	0.796
Amphotericin B	14(9.7)	2(5.3)	0.388	1(3.3)	0(0)	0.650	15(8.6)	2(4.5)	0.368
Capofungin	19(13.2)	5(13.2)	0.995	3(10.0)	0(0)	0.418	22(12.6)	5(11.4)	0.818
Fluconazole	42(29.2)	4(10.5)	0.019	13(43.3)	2(33.3)	0.650	55(31.6)	6(13.6)	0.018
Voriconazole	40(27.8)	16(42.1)	0.089	9(30.0)	3(50.0)	0.343	49(28.2)	19(43.2)	0.055
Capofungin + Fluconazole^#^	14(9.7)	2(5.3)	0.388	**0(0)**	**1(16.7)**	**0.023**	14(8.0)	3(6.8)	0.786
Capofungin + Amphotericin B^#^	**1(0.7)**	**5(13.2)**	**< 0.001**	0(0)	0(0)	-	**1(0.6)**	**5(11.4)**	**< 0.001**
Capofungin + Voriconazole	2(1.4)	0(0)	0.465	3(10.0)	0(0)	0.418	5(2.9)	0(0)	0.255
Fluconazole + Voriconazole	9(6.3)	2(5.3)	0.820	1(3.3)	0(0)	0.65	10(5.7)	2(4.5)	0.755
Fluconazole + Amphotericin B	3(2.1)	2(5.3)	0.286	0(0)	0(0)	-	3(1.7)	2(4.5)	0.264
***Candida*species(*n***, **%)**									
*C. albicans*	64(44.4)	17(44.7)	0.974	3(10.0)	2(33.3)	0.131	67(38.5)	19(43.2)	0.571
non*-C. albicans*	80(55.6)	21(55.3)	1.000	27(90.0)	4(66.7)	0.389	107(61.5)	25(56.8)	0.691
* C. glabrata*	29(20.1)	9(23.7)	0.632	6(20.0)	0(0)	0.230	35(20.1)	9(20.5)	0.960
* C. Tropicalis*	26(18.1)	9(23.7)	0.434	**4(13.3)**	**3(50.0)**	**0.038**	30(17.2)	12(27.3)	0.132
* C. Parapsilosis*	17(11.8)	3(7.9)	0.493	13(43.3)	1(16.7)	0.221	30(17.2)	4(9.1)	0.183
* C.kruseii*	2(1.4)	0(0)	0.465	3(10.0)	0(0)	0.418	5(2.9)	0(0)	0.255
Other *Candida* species	6(4.2)	0(0)	0.201	1(3.3)	0(0)	0.650	7(4.0)	0(0)	0.176
**Wards(*n***, **%)**									
Medical wards	57(39.6)	21(55.3)	0.082	10(33.3)	1(16.7)	0.418	67(38.5)	22(50.0)	0.166
Surgical wards	**45(31.3)**	**5(13.2)**	**0.026**	11(36.7)	2(33.3)	0.877	**56(32.2)**	**7(15.9)**	**0.033**
ICU	42(29.2)	12(31.6)	0.772	9(30.0)	3(50.0)	0.343	51(29.3)	15(34.1)	0.537

*included persistent and non-persistent candidemia patients

^#^Because the sample size was too small, this item was not included in the multivariable logistic regression analysis

^a^ Includes the following diseases: chronic obstructive pulmonary disease and acute respiratory distress syndrome

^b^ Includes the following diseases: cholecystitis, pancreatitis and peritonitis

^c^ Chronic/Acute renal failure is the permanent or sudden and often temporary loss of kidney function with N waste retention and hypourocrinia

^d^ CVC = central venous catheter

^e^a dose equivalent to the prednisone dosage of 0.3 mg/kg/day for at least 14 days

^f^ including: gastrointestinal perforations, severe acute pancreatitis and complex ventral hernia

^g^ICU= intensive care unit

^h^ Neutropenia is the absolute neutrophil count, that is < 500 cells/µl

The mean annual incidence of candidemia was 0.15/1000 admissions, including 0.03/1000 admissions for PC (0.04/1000 at AHSWMU, 0.01/1000 at ZGFPH and 0.02/1000 at SPHNJ) and 0.12/1000 admissions for non-PC (0.14/1000 at AHSWMU, 0.12/1000 at ZGFPH and 0.08/1000 at SPHNJ). According to the *Candida* species, the incidence of the three most commonly isolated *Candida* species in patients with PC were as follows: *C. parapsilosis*, 0.010/1000 admissions; *C. tropicalis*, 0.005/1000 admissions; and *C*. *glabrata*, 0.004/1000 admissions, and that in patients with non-PC was as follows: *C*. *albicans*, 0.056/1000 admissions; *C*. *glabrata*, 0.026/1000 admissions and *C*. *tropicalis*, 0.024/1000 admissions. The detailed data in the three hospitals are shown in Supplementary Table S1.

**Fig. 2:**
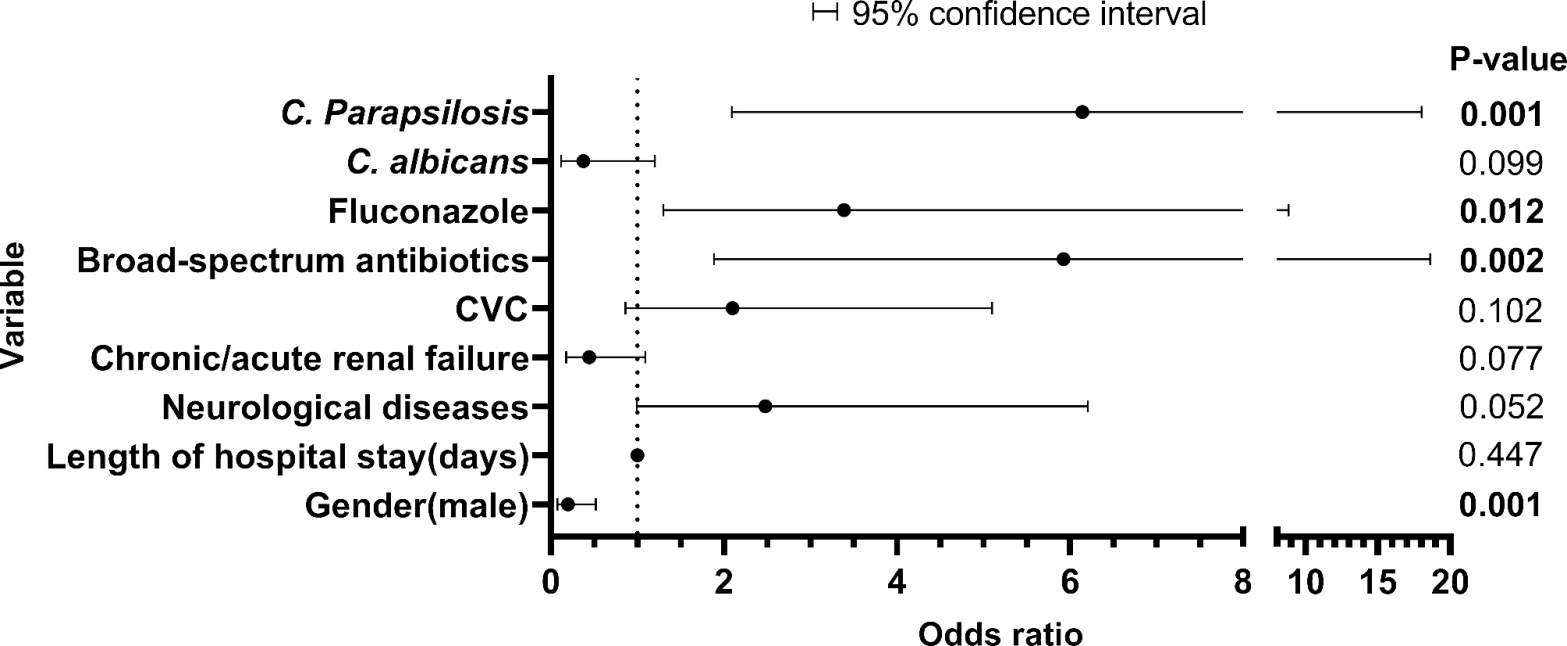
Factors associated with the formation of PC by multivariate analysis FootNote: Length of hospital stay(days)( 95% CI: 0.996–1.009)

The most common species among all *Candida* species isolates were *C*. *albicans* (39.4%), followed by *C*. *glabrata* (20.2%), *C*. *tropicalis* (19.3%), *C. parapsilosis* (15.6%), *C. krusei* (2.3%) and others (3.2%). The distribution of *Candida* species in patients with PC and non-PC is shown in Table 1. *C. parapsilosis* was the predominant species in patients with PC (38.9%), whereas *C*. *albicans* was the main species in patients with non-PC (44.5%). The distribution of *Candida* species in patients with PC and non-PC in surgical, medical and ICU wards is shown in Fig. 1. The detailed data from the three hospitals are shown in Supplementary Table S2.

The results of in vitro susceptibility testing of *Candida* strain isolates are summarized in Table 2. All isolates were highly susceptible to AMB (99.5%) and 5-FC (99.0%), and the resistance rate of ITR, VRC and FCA was 18.6%, 15.2%, and 14.1%, respectively. *C. tropicalis* had the highest antifungal agent resistance rate among the *Candida* species, which was resistant to FCA (36.6%), ITR (36.6%) and VRC (39.0%). The activity of antifungal agents against the *Candida* species was not significantly different between patients with PC and non-PC (*P* > 0.05). The detailed data are shown in Table 2.

The all-cause mortality rate in the 218 patients was 26.1% (57/218). The 7-day and 30-day mortality rates were 10.1% (22/218) and 20.2% (44/218), respectively. The 7-day mortality rate for patients with PC and non-PC was 2.8% (1/36) and 11.5% (21/182) and the 30-day mortality rate was 16.7% (6/36) and 20.9% (38/182), respectively. The 30-day mortality rate for medical wards, surgical wards, and ICU wards in patients with PC and non-PC was 9.1% (1/11), 15.4% (2/13) and 16.7% (2/12), and 26.9% (21/78), 10.0% (5/50) and 22.2% (12/54), respectively.

**Fig. 3:**
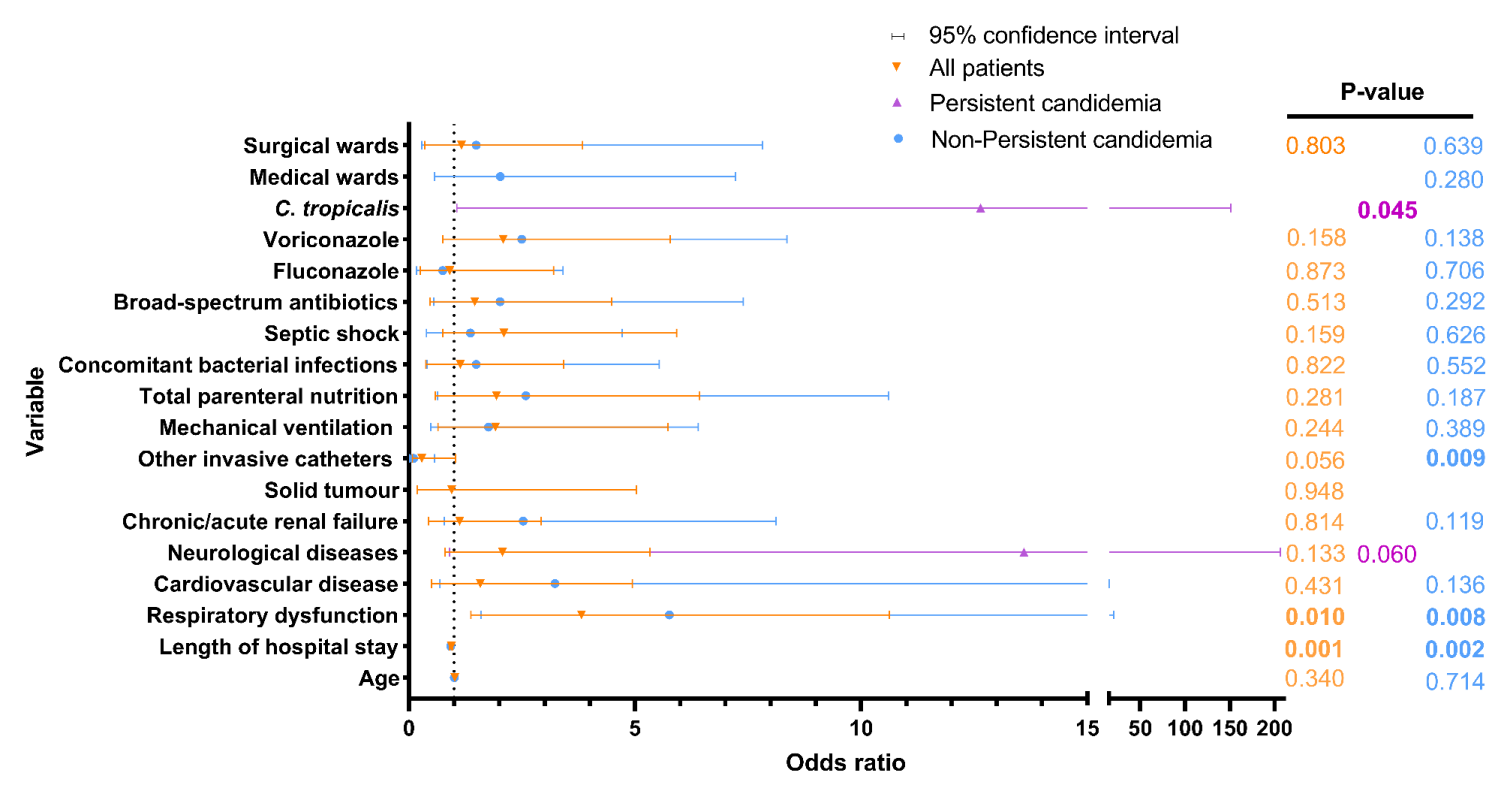
Factors associated with 30-day mortality by multivariate analysis FootNote: Non-Persistent candidemia: Age (95%CI: 0.975–1.038), Length of hospital stay(days)(95% CI: 0.880–0.973). All patients: Age (95%CI: 0.986–1.041), Length of hospital stay(days)(95% CI: 0.905–0.973)

The univariate predictors of poor outcomes due to PC are shown in Table 1. The results of the multivariate analysis showed that use of broad-spectrum antibiotics (OR, 5.925; 95% CI, 1.886–18.616, *P* = 0.002) and FCA (OR, 3.389; 95% CI, 1.302–8.820, *P* = 0.012), and *C. parapsilosis* infection (OR, 6.143; 95% CI, 2.093–18.031, *P* = 0.001) were independent risk factors for PC, sex (male) (OR, 0.199; 95% CI, 0.077–0.518, P = 0.001) was the protective factor for PC (Fig. 2 and Supplementary Table S3). The variable associated with 30-day mortality for adult patients with PC was *C. tropicalis*, and the variables for those with non-PC were age, length of hospital stay, respiratory dysfunction, cardiovascular disease, chronic/acute renal failure, other invasive catheters, mechanical ventilation, total parenteral nutrition, concomitant bacterial infections, septic shock, use of broad-spectrum antibiotics and FCA, and surgical wards (Table 3). The results of the multivariate analysis associated with the 30-day mortality in patients with PC and non-PC are listed in Fig. 3 and Supplementary Table S4. *C. tropicalis* bloodstream infection was the only independent predictor of 30-day mortality in patients with PC (OR, 12.642; 95% CI, 1.059–150.951; *P* = 0.045). The length of hospital stay(OR, 0.925; 95% CI, 0.880–0.973, P = 0.002) and respiratory dysfunction (OR, 5.763; 95% CI, 1.592–20.864, P = 0.008) were independent predictors of 30-day mortality in patients with non-PC (Fig. 3 and Supplementary Table S4). Other invasive catheters were only the protective factor for 30-day mortality in patients with non-PC (OR, 0.104; 95% CI, 0.019–0.568, *P* = 0.009).

## Discussion

This was a 6-year multicenter retrospective study of PC and non-PC in three tertiary teaching hospitals in Southwest China. We analyzed the clinical characteristics, including the demographics, underlying comorbidities, risk factors, distribution of *Candida* species, antifungal therapy, antifungal agent susceptibility results, department of admission and patient outcomes, as well as epidemiologically compared patients with PC and non-PC.

**Table 4:** Protective factor and predictors of PC and 30-day mortality in other studies from 2012–2021

Authors	Country or region	study period	study design	samples	No of samples	Protective factor for PC	Independent risk factors for PC	Predictors of 30-day mortality	Reference
Ala-Houhala et al.	Finland	2007–2016	Retrospective, cohort single-center (two hospital district )study	Adult (75 persistent and 151 non-persistent candidemia patients)	226	early source control	CVC, metastatic infection foci, ineffective empirical treatment	-	4
Agnelli .et al	Spain	2010–2018	Retrospective, observational, single-center study	Adult (35 persistent and 220 non-persistent candidemia patients)	255	-	undetected site of infection	-	15
Kang et al.	South Korea	2007–2014	Retrospective, study (2 tertiary general hospitals)	Adult (72 persistent and 117 non-persistent candidemia patients)	189		CVC, longer hospital stay, severe sepsis	*C. tropicalis*, Septic shock, Corticosteroid within the past 30 days	9
Fu et al.	China	2012–2015	Retrospective, observational, single-center study	Neonates (28 persistent and 20 non-persistent candidemia patients)	48		Intubation and Mechanical ventilation		16
Hammoud et al.	Kuwait	2007–2010	Retrospective, observational, single-center study	Neonates (54 persistent and 34 non-persistent candidemia patients)	88		female gender, presence of CVC and platelet count < 50*10^9^/L		5
Robinson et al.	USA	2000–2010	Retrospective, observational, single-center study hospitals)	Neonates (9 persistent and 26 non-persistent candidemia patients)	37		antifungal therapy was started > 1 day from positive blood culture		21
This study	China	2016–2021	Retrospective, observational, multicentre, cohort study	Adult (36 persistent and 182 non-persistent candidemia patients)	218	male	the use of broad-spectrum antibiotics and fluconazole	*Candida tropicalis* bloodstream infection	This study

CVC: central venous catheter

Our data showed no significant difference in age, department of admission, and 30-day mortality between patients with PC and non-PC (*P* > 0.05). Our data were consistent with the findings of other studies conducted in adult patients with PC and non-PC [11, 18]. The incidence rate in adult patients with PC (0.03/1000 admissions) was significantly lower than in infant patients(5.5/1000 admissions) [19], This may be related to the clinical characteristics of the patients, and the infant’s immune system is even worse [20]. Meanwhile, the proportion of underlying comorbidities in PC and non-PC, except for chronic/acute renal failure, was not significantly different (*P* > 0.05). The proportion of chronic/acute renal failure was lower in patients with PC than in those with non-PC (*P* < 0.05) (Fig. 2 and Supplementary Table S3). Among the risk factors, only CVC had higher risks in patients with PC than those with non-PC (*P* < 0.05), and the proportion of other risk factors was similar for both patients (*P* > 0.05) (Fig. 2 and Supplementary Table S3), consistent with previous studies [11, 18]. In therapy, the proportion of use of broad-spectrum antibiotics, FCA and capofungin + VRC were higher in patients with PC than in patients with non-PC (*P* < 0.05). After *Candida* was identified in blood, VRC and FCA were used as first-line drugs against the *Candida* infection, which may be related to the high sensitivity of *Candida* species to azole antifungal drugs (Table 2). Meanwhile, 21.1% (46/218) patients were treated with the combination drug for *Candida spp*., possibly because of the drug resistance of *Candida* or the severity of the patient’s condition. Although echinococcus is the first-line therapy of candidemia, caspofungin was the most used echinocandin drug in many countries [21, 22], however, caspofungin was also a higher risk of inducing FKS mutations in comparison to other echinocandins [21, 23], leading to gradual increase in the resistance rate of caspofungin. There are no susceptibility tests for echinococcus in our region, which may be the reason why clinicians were less likely to choose echinococcus as an first-line agent.

Our data showed that the number of female patients with PC was higher than that with non-PC, which was different from the results of other studies. However, the proportion of men was similar to that in other studies [4, 11, 18], however, the proportion of female were similar to the result of infants study in China [19]. Moreover, the present study showed that the length of hospital stay was longer for patients with PC than for those with non-PC (*P* = 0.016), which was consistent with the reports of other studies [4]. The patients with PC were hospitalized mostly in surgical wards, and those with non-PC mostly in medical wards, which was similar to other studies that reported hospitalization in Spain [18], and different from those in Finland [4]. This phenomenon may be related to the demographic characteristics of the inpatients in different hospitals or regions. According to our study, *C. albicans* was the most common cause of candidemia in the whole region, but the proportion of non–*C. albicans* infections was higher than that of *C. albicans* infections in patients with PC. Moreover, the proportion of *C. parapsilosis* in surgical, medical and ICU wards was the highest for patients with PC, which was different from other studies in other countries [4, 11, 18]. This may be due to the demographic characteristics of the patients in different hospitals or regions, or few statistical samples (36 cases of PC).

Our data showed that the mean incidence of PC was 0.03 episodes/1000 admissions from 2016 to 2021. However, the incidence rate was different in different hospitals [4, 11, 18, 24], which was mainly related to the diagnosis and treatment characteristics of hospitals and the basic conditions of patients. Further, 36 patients (13.7%) fulfilled the definition of PC, which was higher than that reported by Kang et al. [11], and less than that reported by Ala-Houhala et al. [4]. The 30-day mortality in this study was similar to that in some hospitals in other countries [4], but lower than that in some other hospitals in other countries [11]. The reason may be that the most persistent *Candida* infections are caused by *C. parapsilosis* in this region, and they are sensitive to all antifungal agents (Table 2), which may also be one of the reasons for the low mortality rate of persistent *Candida* infection in this area. The 30-day mortality in ICU wards was the highest among patients with PC and non-PC, which may be related to the severity of underlying diseases in ICU patients, and was consistent with other studies.

Resistance to FCA, ITR and VRC was common in *C. albicans* and non-C. *albicans* species (Table 2). In our study, AMB and 5-FC were highly active against all *Candida* species. In patients with PC, the resistance rate of ITR was the highest, and the resistance rates of ITR and FCA were higher than those in patients with non-PC. However, the resistance rate of *Candida* species was not significantly different between patients with PC and non-PC (*P* > 0.05), the resistance rate of *Candida* species was not associated with the development of persistent candidemia, which is inconsistent with the result of another study [10]. Moreover, FCA was highly active against all *Candida* species in patients with PC and non-PC and could be used in patients with candidemia as a first-line agent. In the whole region, the resistance rate to azole was similar to those reported in other regions and countries [25–27], but *C. tropicalis* and *C. albicans* showed high resistance to azole antifungal drugs in patients with PC in this region. The mechanism of drug resistance will be researched in later studies. This may be related to the long-term use of azole antifungal drugs in patients with persistent *Candida* infection. Therefore, the antifungal susceptibility of the strains isolated from patients with persistent *Candida* infection needs to be analyzed so as to guide clinicians to choose antifungal drugs reasonably and avoid the continuous increase of drug resistance.

In this study, we analyzed the risk factors in adult patients with PC and non-PC using multifactorial regression, and the results revealed that use of broad-spectrum antibiotics(OR: 5.925) and FCA(OR: 3.389), and *C. parapsilosis* infection(OR: 6.143) were independent risk factors for patients with PC, and sex (male) (OR: 0.199) was the protective factor for PC, which was different from the results of other studies, the other studies have showed that CVC(OR:2.71), metastatic infection foci(OR:3.60), ineffective empirical treatment (OR: 3.31) and unsuspected sites of infection (OR: 4.28) were independent risk factors for patients with PC [4, 11, 18]. The age, length of hospital stay, respiratory dysfunction, cardiovascular disease, chronic/acute renal failure, other invasive catheters, mechanical ventilation, total parenteral nutrition, concomitant bacterial infections, septic shock, use of broad-spectrum antibiotics such as FCA and Capofungin + AMB, and surgical wards were the common predictors of mortality in the univariate analysis (*P* < 0.05) in patients with non-PC, and the univariate predictors of poor outcomes in patients with PC were less than those in patients with non-PC (1 vs. 13 predictors), as shown in Table 3. *C. tropicalis* bloodstream infection was only the common predictor of mortality in the univariate analysis (*P* < 0.05) in patients with PC; meanwhile, it was also the only independent risk factor for 30-day mortality (OR:12.642). The reason may be because *C. tropicalis* has a high resistance to the antifungal drugs of azole, leading to the failure of treatment in patients with *C. tropicalis* infection, finally, the death of patients, which was consistent with the findings of another study in South Korea (OR: 4.12) [11]. Respiratory dysfunction (OR: 5.763) was independent predictors of 30-day mortality in this study, however, some other studies have reported that corticosteroid within past 30 days (OR:5.31) and Septic shock (OR: 5.81) were independent predictors of 30-day mortality. The length of hospital stays (OR: 0.925) and other invasive catheters (OR: 0.104) were the protective factors for 30-day mortality in patients with non-PC. Previous studies have reported respiratory dysfunction(OR: 22.57) as an independent predictor [28]. However, the length of hospital stay (OR: 0.89) and other invasive catheters (OR: 0.04) reported here have rarely been reported in other studies, possibly because the demographic characteristics, underlying diseases and risk factors of the patients in our study were different from those in other studies. This may be why the independent predictors and protective factors in this study differed from those in other studies (see Table 4).

This study has two potential limitations. First, all *Candida* strain isolates were tested for antifungal susceptibility using ATB FUNGUS 3 kit (bioMérieux, France) in all three hospitals, the kit did not contain echinococcins, we only had data on the use of echinococcins, but no data on drug sensitivity. Second, although we conducted a multicenter retrospective study, our total sample size was still smaller. Our data may be affected by the insufficient sample size. Therefore, the results may not be generalizable to patients with persistent candidemia in other regions of China.

## Conclusions

*C. albicans* was the main *Candida* species, but *C. parapsilosis* has become the most common species in PC in the study region. FCA was the main antifungal drug for patients with PC and a prophylaxis antifungal therapy. AMB and 5-FC were highly active against all *Candida* species. The morbidity and mortality rates in patients with PC and non-PC in this region were lower than those in other regions. The length of hospital stay and respiratory dysfunction were independent predictors of 30-day mortality in adult patients with non-PC. *C. tropicalis* infection was the independent risk factor for the 30-day mortality in adult patients with PC. This study provides reference data of epidemiological and antifungal drug susceptibility for the prevention and treatment of adult patients with PC in other hospitals in China.

## Electronic supplementary material

Below is the link to the electronic supplementary material.

Supplementary Material 1

Supplementary Material 2

Supplementary Material 3

Supplementary Material 4

## Data Availability

The datasets used and/or analysed during the current study available from the corresponding author on reasonable request.

## Abbreviations

PC: persistent candidemia
BSI: bloodstream infection
ICU: intensive care unit
USA: United States of America
ATCC: American type culture collection
MALDI-TOF MS: Matrix-assisted laser desorption/ionization-time of flight mass spectroscopy
FCA: fluconazole
ITR: itraconazole
AMB: amphotericin B
VRC: voriconazole
5-FC: flucytosine
CVC: central venous catheter
MIC: minimal inhibitory concentration
OR: odds ratio
CI: confidence interval
SD: standard deviation.
