# Supplementary material for: Epidemiology, antifungal susceptibility, risk factors, and mortality of persistent candidemia in adult patients in China: a 6-year multicenter retrospective study
Source: BMC Infect Dis. 2023 Jun 1;23:369. doi: 10.1186/s12879-023-08241-9 (PMC10233919; doi:10.1186/s12879-023-08241-9)
Supplement: Supplementary file 1 — Supplementary Material 1 [file 12879_2023_8241_MOESM1_ESM.docx]

Table S1. Incidence of *Candida* species in three hospitals

^*^ The second people's Hospital of Neijiang has no data in 2016.

| year | Incidence (episodes/1,000 admissions) | | | | | | | | |
| --- | --- | --- | --- | --- | --- | --- | --- | --- | --- |
|  | The Affiliated Hospital of SWMU^#^ | | | Zigong fourth people's Hospital | | | The second people's Hospital of Neijiang^*^ | | |
|  | total | persistent | non-  persistent | total | persistent | non-  persistent | total | persistent | non-  persistent |
| 2016 | 0.16 | 0.04 | 0.12 | 0.18 | 0.02 | 0.16 | - | - | - |
| 2017 | 0.16 | 0.02 | 0.14 | 0.12 | 0.06 | 0.06 | 0.05 | 0 | 0.05 |
| 2018 | 0.13 | 0 | 0.13 | 0.13 | 0.02 | 0.11 | 0.18 | 0.07 | 0.11 |
| 2019 | 0.23 | 0.07 | 0.16 | 0.16 | 0 | 0.16 | 0.02 | 0 | 0.02 |
| 2020 | 0.15 | 0.03 | 0.12 | 0.15 | 0 | 0.15 | 0.18 | 0 | 0.18 |
| 2021 | 0.21 | 0.04 | 0.17 | 0.08 | 0 | 0.08 | 0.09 | 0.02 | 0.07 |
| Mean annual incidence | 0.18 | 0.04 | 0.14 | 0.13 | 0.01 | 0.12 | 0.10 | 0.02 | 0.08 |

^#^ The Affiliated Hospital of Southwest Medical University.
