# Supplementary material for: Epidemiology, antifungal susceptibility, risk factors, and mortality of persistent candidemia in adult patients in China: a 6-year multicenter retrospective study
Source: BMC Infect Dis. 2023 Jun 1;23:369. doi: 10.1186/s12879-023-08241-9 (PMC10233919; doi:10.1186/s12879-023-08241-9)
Supplement: Supplementary file 2 — Supplementary Material 2 [file 12879_2023_8241_MOESM2_ESM.docx]

Table S2.Distribution of *Candida* species in three hospitals.

| Distribution n(%) | *Candida* species (persistent/non-persistent) | | | | | | |
| --- | --- | --- | --- | --- | --- | --- | --- |
|  | Total | *C. albicans* | *C. glabrata* | *C. tropicalis* | *C. parapsilosis* | *C. krusei* | *Others** |
| The Affiliated Hospital of SWMU^#^(138, 63.3%) | 26(72.2)/112(61.5) | 3(11.5)/47(42) | 5(19.2)/26(23.2) | 5(19.2)/26(23.2) | 10(38.5)/8(7.1) | 3(11.5)/2(1.8) | 0(0)/3(2.7) |
| Age, years, mean (SD) | 58.4(16.3)/  56.5(18.1) | 70.7(19.3)/  58.3(17.0) | 64.0(15.3)/  61.4(17.0) | 58.4(8.6)/  51.5(18.3) | 55.9(19.6)/  46.8(21.0) | 45.3(3.5)/  44.5.(31.8) | -/64.7(18.6) |
| Male (76, 55.1%) | 11(42.3)/65(58.0) | 1(9.1)/24(36.9) | 1(9.1)/16(24.6) | 2(18.2)/16(24.6) | 5(45.5)/6(9.2) | 2(18.2)/1(1.5) | 0(0)/2(3.1) |
| Female (62, 44.9%) | 15(57.7)/47(42.0) | 2(13.3)/23(48.9) | 4(26.7)/10(21.3) | 3(20)/10(21.3) | 5(33.3)/2(4.3) | 1(6.7)/1(2.1) | 0(0)/1(2.1) |
| Medical (65, 47.1% ) | 7(26.9)/58(51.8) | 0(0)/23(39.7) | 2(28.6)/14(24.1) | 4(57.1)/16(27.6) | 0(0)/4(6.9) | 1(14.3)/0(0) | 0(0)/1(1.7) |
| Surgical (34, 24.6% ) | 9(34.6)/25(22.3) | 1(11.1)/10(40) | 1(11.1)/6(24) | 1(11.1)/4(16) | 6(66.7)/4(16) | 0(0)/0(0) | 0(0)/1(4) |
| ICU (39, 28.3% ) | 10(38.5)/29(25.9) | 2(20)/14(48.3) | 2(20)/6(20.7) | 0(0)/6(20.7) | 4(40)/0(0) | 2(20)/2(6.9) | 0(0)/1(3.4) |
| Zigong fourth people's Hospital (58, 26.6%) | 6(16.7)/52(28.6) | 1(16.7)/28(53.8) | 1(16.7)/10(19.2) | 1(16.7)/2(3.8) | 2(33.3)/10(19.2) | 0(0)/0(0) | 1(16.7)/2(3.8) |
| Age, years, mean (SD) | 63.8(12.9)/  67.5(12.3) | 62.0(0.0)/  69.6(10.5) | 54.0(0.0)/  67.3(12.7) | 69.0(0.0)/  51.0(8.5) | 65.5(26.2)/  68.5(13.9) | - | 67.0(0.0)/  49.5(13.4) |
| Male (37, 63.8%) | 3(50)/34(65.4) | 0(0)/15(44.1) | 1(33.3)/8(23.5) | 0(0)/1(2.9) | 1(33.3)/8(23.5) | 0(0)/0(0) | 1(33.3)/2(5.9) |
| Female (21, 36.2%) | 3(50)/18(34.6) | 1(33.3)/13(72.2) | 0(0)/2(11.1) | 1(33.3)/1(5.6) | 1(33.3)/2(11.1) | 0(0)/0(0) | 0(0)/0(0) |
| Medical (14, 24.1%) | 2(33.3)/12(23.1) | 0(0)/7(58.3) | 0(0)/4(33.3) | 0(0)/0(0) | 2(100)/1(8.3) | 0(0)/0(0) | 0(0)/0(0) |
| Surgical (24, 41.4%) | 2(33.3)/22(42.3) | 0(0)/14(63.6) | 1(50)/4(18.2) | 0(0)/0(0) | 0(0)/3(13.6) | 0(0)/0(0) | 1(50)/1(4.5) |
| ICU (20, 34.5%) | 2(33.3)/18(34.6) | 1(50)/7(38.9) | 0(0)/2(11.1) | 1(50)/2(11.1) | 0(0)/6(33.3) | 0(0)/0(0) | 0(0)/1(5.6) |
| The second people's Hospital of Neijiang (22, 10.1%) | 4(11.1)/18(9.9) | 1(25)/6(33.3) | 0(0)/2(11.1) | 1(25)/7(38.9) | 2(50)/2(11.1) | 0(0)/0(0) | 0(0)/1(5.6) |
| Age, years, mean (SD) | 64.8(6.9)/  58.3(16.3) | 75.0(0)/  52.2(12.6) | -/78.0(2.8) | 60.0(0)/  59.1(14.6) | 62(0.0)/  71.0(2.8) | - | -/25.0(0.0) |
| Male (18, 81.8%) | 2(50.0)/16(88.9) | 1(50)/6(37.5) | 0(0)/2(12.5) | 1(50)/5(31.3) | 0(0)/2(12.5) | 0(0)/0(0) | 0(0)/1(6.3) |
| Female (4, 18.2%) | 2(50.0)/2(11.1) | 0(0)/0(0) | 0(0)/0(0) | 0(0)/2(100) | 2(100)/0(0) | 0(0)/0(0) | 0(0)/0(0) |
| Medical (10, 45.5%) | 2(50.0)/8(44.4) | 0(0)/2(25) | 0(0)/0(0) | 0(0)/4(50) | 2(100)/1(12.5) | 0(0)/0(0) | 0(0)/1(12.5) |
| Surgical (5, 22.7%) | 2(50.0)/3(16.7) | 1(50)/2(66.7) | 0(0)/1(33.3) | 1(50)/0(0) | 0(0)/0(0) | 0(0)/0(0) | 0(0)/0(0) |
| ICU (7, 31.8%) | 0(0)/7(38.9) | 0(0)/2(28.6) | 0(0)/1(14.3) | 0(0)/3(42.9) | 0(0)/1(14.3) | 0(0)/0(0) | 0(0)/0(0) |
| Three Hospitals (218,100%) | 36(100)/182(100) | 5(13.9)/81(44.5) | 6(16.7)/38(20.9) | 7(19.4)/35(19.2) | 14(38.9)/20(11) | 3(8.3)/2(1.1) | 1(2.8)/6(3.3) |
| Age, years, mean (SD) | 60.0(15.0)/  59.8(17.1) | 59.8(14.4)/  61.7(15.8) | 62.3(14.3)/  63.8(15.9) | 60.1(8.0)/  53.0(17.2) | 58.1(18.3)/  60.1(19.5) | 45.3(3.5)/  44.5(31.8) | 67.0(0)/  53.0(20.4) |
| Male(131, 60.1%) | 16(44.4)/115(63.2) | 2(12.5)/45(39.1) | 2(12.5)/26(22.6) | 3(18.8)/22(19.1) | 6(37.5)/16(13.9) | 2(12.5)/1(0.9) | 1(6.3)/5(4.3) |
| Female(87, 39.9%) | 20(55.6)/67(36.8) | 3(15)/36(53.7) | 4(20)/12(17.9) | 4(20)/13(19.4) | 8(40)/4(6) | 1(5)/1(1.5) | 0(0)/1(1.5) |
| Medical (89, 40.8%) | 11(30.6)/78(42.9) | 0(0)/32(41.0) | 2(18.2)/18(23.1) | 4(36.4)/20(25.6) | 4(36.4)/6(7.7) | 1(9.1)/0(0) | 0(0)/2(2.6) |
| Surgical (63,28.9%) | 13(36.1)/50(27.5) | 2(15.4)/26(52.0) | 2(15.4)/11(22.0) | 2(15.4)/4(8.0) | 6(46.2)/7(14.0) | 0(0)/0(0) | 1(7.7)/2(4) |
| ICU (66, 30.3%) | 12(33.3)/54(29.7) | 3(25)/23(42.6) | 2(16.7)/9(16.7) | 1(8.3)/11(20.4) | 4(33.3)/7(13.0) | 2(16.7)/2(3.7) | 0(0)/2(3.7) |

*Others include *C. guilliermondii (2), C. famata (2), C. dubliniensis (1), C. haemulonii (1) and C. inconspicua (1)*.

# The Affiliated Hospital of Southwest Medical University.
