# Supplementary material for: Epidemiology, antifungal susceptibility, risk factors, and mortality of persistent candidemia in adult patients in China: a 6-year multicenter retrospective study
Source: BMC Infect Dis. 2023 Jun 1;23:369. doi: 10.1186/s12879-023-08241-9 (PMC10233919; doi:10.1186/s12879-023-08241-9)
Supplement: Supplementary file 3 — Supplementary Material 3 [file 12879_2023_8241_MOESM3_ESM.docx]

Table S3. Factors associated with the formation of PC by multivariate analysis

| Variable | Odds ratio | 95% confidence interval | *P*-value |
| --- | --- | --- | --- |
| **Gender(male)** | **0.199** | **0.077-0.518** | **0.001** |
| Length of hospital stay(days) | 1.002 | 0.996-1.009 | 0.447 |
| Neurological diseases | 2.482 | 0.993-6.206 | 0.052 |
| Chronic/acute renal failure | 0.443 | 0.18-1.091 | 0.077 |
| CVC | 2.099 | 0.864-5.101 | 0.102 |
| **Broad-spectrum antibiotics** | **5.925** | **1.886-18.616** | **0.002** |
| **Fluconazole** | **3.389** | **1.302-8.82** | **0.012** |
| *C. albicans* | 0.379 | 0.12-1.202 | 0.099 |
| ***C. Parapsilosis*** | **6.143** | **2.093-18.031** | **0.001** |

*Because the number was very small, the Capofungin+Voriconazole and *C.kruseii* were not included in the multivariable logistic regression analysis.
