# Supplementary material for: Epidemiology, antifungal susceptibility, risk factors, and mortality of persistent candidemia in adult patients in China: a 6-year multicenter retrospective study
Source: BMC Infect Dis. 2023 Jun 1;23:369. doi: 10.1186/s12879-023-08241-9 (PMC10233919; doi:10.1186/s12879-023-08241-9)
Supplement: Supplementary file 4 — Supplementary Material 4 [file 12879_2023_8241_MOESM4_ESM.docx]

Table S4. Factors associated with 30-day mortality by multivariate analysis

| Variable | Non-Persistent candidemia | | | Persistent candidemia* | | |  | All patients |  |
| --- | --- | --- | --- | --- | --- | --- | --- | --- | --- |
|  | OR | 95% CI | *P* | OR | 95% CI | *P* | OR | 95%CI | *P* |
| Age | 1.006 | 0.975-1.038 | 0.714 |  |  |  | 1.013 | 0.986-1.041 | 0.34 |
| **Length of hospital stay** | **0.925** | **0.880-0.973** | **0.002** |  |  |  | **0.938** | **0.905-0.973** | **0.001** |
| **Respiratory dysfunction** | **5.763** | **1.592-20.864** | **0.008** |  |  |  | **3.819** | **1.373-10.622** | **0.010** |
| Cardiovascular disease | 3.228 | 0.692-15.06 | 0.136 |  |  |  | 1.581 | 0.506-4.939 | 0.431 |
| Neurological diseases |  |  |  | 13.604 | 0.898-205.997 | 0.060 | 2.067 | 0.801-5.329 | 0.133 |
| Chronic/acute renal failure | 2.529 | 0.788-8.120 | 0.119 |  |  |  | 1.122 | 0.430-2.927 | 0.814 |
| Solid tumour |  |  |  |  |  |  | 0.946 | 0.178-5.033 | 0.948 |
| **Other invasive catheters** | **0.104** | **0.019-0.568** | **0.009** |  |  |  | 0.283 | 0.078-1.031 | 0.056 |
| Mechanical ventilation | 1.763 | 0.485-6.402 | 0.389 |  |  |  | 1.916 | 0.641-5.727 | 0.244 |
| Total parenteral nutrition | 2.587 | 0.631-10.604 | 0.187 |  |  |  | 1.935 | 0.582-6.431 | 0.281 |
| Concomitant bacterial infections | 1.489 | 0.400-5.538 | 0.552 |  |  |  | 1.135 | 0.377-3.420 | 0.822 |
| Septic shock | 1.361 | 0.394-4.709 | 0.626 |  |  |  | 2.104 | 0.748-5.920 | 0.159 |
| Broad-spectrum antibiotics | 2.014 | 0.548-7.394 | 0.292 |  |  |  | 1.455 | 0.472-4.484 | 0.513 |
| Fluconazole | 0.747 | 0.163-3.414 | 0.706 |  |  |  | 0.902 | 0.254-3.199 | 0.873 |
| Voriconazole | 2.495 | 0.744-8.360 | 0.138 |  |  |  | 2.084 | 0.752-5.777 | 0.158 |
| *C. tropicalis* |  |  |  | **12.642** | **1.059-150.951** | **0.045** |  |  |  |
| Medical wards | 2.019 | 0.564-7.223 | 0.280 |  |  |  |  |  |  |
| Surgical wards | 1.487 | 0.283-7.815 | 0.639 |  |  |  | 1.164 | 0.353-3.844 | 0.803 |

ICU= intensive care unit, OR=odds ratio, CI=confidence interval
